# Supplementary material for: MetaRibo-Seq measures translation in microbiomes
Source: Nat Commun. 2020 Jun 29;11:3268. doi: 10.1038/s41467-020-17081-z (PMC7324362; doi:10.1038/s41467-020-17081-z)
Supplement: Supplementary file 10 — Supplementary Data 7 [file 41467_2020_17081_MOESM10_ESM.zip › File2/Confidence_High_Taxonomy/24283_out.krona.html]

Javascript must be enabled to view this page.

members
magnitude
magnitudeUnassigned
count
unassigned
taxon
rank

24283\_out

1

superkingdom
2
1

phylum
1224
1

1
68525
subphylum

class
29547
1

1
213849
order

family
1
72293

genus
209
1

1
210

SRS143509\_contig\_number\_contig-100\_537.42652
species
